# Israeli dentists’ knowledge, attitudes, and practices regarding smoking cessation care

**DOI:** 10.1186/s13584-024-00653-5

**Published:** 2024-11-11

**Authors:** Tamar Vishnevsky, Tal Aperman-Itzhak, Itzhak Tayeb, Yael Bar-Zeev

**Affiliations:** 1 Braun School of Public Health and Community Medicine, The Faculty of Medicine, Hebrew University of Jerusalem-Hadassah Medical Center, Jerusalem, Israel; 2 The Maurice and Gabriela Goldschleger School of Dental Medicine, Faculty of Medicine, Tel Aviv University, Tel Aviv, Israel; 3 Hadassah Medical Center - Mount Scopus Campus, Jerusalem, Israel

**Keywords:** Dentists, Smoking cessation care, Theoretical domains framework, 5A’s performance

## Abstract

**Background:**

The 5A’s model for brief smoking cessation care (SCC) is recommended for dentists to reduce the negative impacts of smoking on oral health. This study investigates Israeli dentists' adherence to the 5A’s guidelines and explores factors influencing their knowledge, attitudes, and practices.

**Methods:**

An online cross-sectional survey was conducted among Israeli dentists during June–August 2020. The questionnaire included sociodemographic, smoking, and professional characteristics; knowledge (10 true/false statements); attitudes regarding SCC [based on the Theoretical Domains Framework (TDF)] using 13 statements (1–5 Likert scale), for a composite mean attitude score; and 5A’s performance (1–5 Likert scale, never to always). Two primary outcomes were analysed: (1) performing all of the 5A’s ‘often or always’; and (2) performing ‘always’ the first two steps (“Ask” and “Advise”). Multivariable logistic regression explored the association between the various characteristics and the primary outcomes.

**Results:**

Overall, n = 410 responded. Mean knowledge score was 2.58 (SD = 1.51). Mean attitude score was 2.65 (SD = 0.60). Performance of all 5A’s was low with 14.1% (n = 57) reporting completing all 5A’s ‘often or always’, while 34.1% (n = 139) reported ‘always’ performing ‘Ask’ and ‘Advise’. Specialists had better odds of ‘often or always’ performing the 5A’s (adjusted OR = 2.01, p = .022) and ‘always’ performing ‘Ask and Advise’ (adjusted OR = 1.71, p = .022).

**Conclusions:**

This study highlights the insufficient performance of SCC among Israeli dentists, revealing gaps in knowledge and attitudes related to SCC. Various measures, such as training, automatic referral systems, and integrating SCC as quality measures, may improve SCC provision among Israeli dentists.

**Supplementary Information:**

The online version contains supplementary material available at 10.1186/s13584-024-00653-5.

## Introduction

Smoking has a significant adverse impact on oral health [1]. Oral and oropharyngeal cancers are two to five times more likely to develop in cigarette smokers than in non-smokers [1] and are correlated with number of pack years smoked [1, 2].

Smoking cessation reduces the negative impacts of smoking on oral health [3], including reducing risk of oral cancers [4] and benign diseases such as periodontitis [5], periodontal therapy complications [6] and tooth discoloration [7]. It also improves oral health-related quality of life, such as enhancing taste [8, 9]. Smokers tend to demonstrate inferior periodontal treatment outcomes compared with nonsmokers [10]. Therefore, smoking cessation care (SCC) should be promoted by dentists and integrated routinely within dental care [10]. Periodontists may play a more significant role than general dentists in this regard, as their specialty inherently involves long-term and continual patient care [11]. Additionally, a study found that public sector dentists were more knowledgeable, skilled, and positive about offering smoking cessation counseling than private dentists. However, they also reported more frequently feeling that they lacked sufficient time and support systems for follow-up compared to their private counterparts [12].

Provision of SCC in the oral health setting is both feasible and effective [13], and dentist-delivered SCC was found to be well accepted by participants [14]. In Israel, about 20% of the adult population smokes, leading to approximately 8000 deaths per year [15]. All of the Health Maintenance Organizations provide free behavioral therapy combined with subsidized pharmacotherapy for smoking cessation. However, only 2.5% of Israeli smokers utilized this service in 2022 [15]. Currently, the Israeli Dental Association (IDA) does not have any clinical guidelines regarding the provision of SCC. However, clinical guidelines from other countries, such as the USA, UK and Australia, include recommendations regarding SCC provision [16–19]. The American Dental Association (ADA) guidelines on the dentist’s role in preventing tobacco use, states that dentists can help their patients quit smoking by consistently identifying which patients smoke, advising them to quit, and offering information about cessation treatment. Specifically, the ADA recommends using the “the 5A’s” when interacting with patients who smoke [13, 18, 20]: (1) Ask—Identify and document smoking status for every patient at every visit; (2) Advise—In a clear, strong, and personalized manner, urge every smoker to quit; (3) Assess—Is the smoker willing to make a quit attempt at this time?; (4) Assist—For the patient willing to make a quit attempt, use counseling and pharmacotherapy to help him or her quit; and (5) Arrange—Schedule follow-up contact, in person or by telephone, preferably within the first week after the quit date.

Training dentists to deliver brief smoking cessation interventions such as the 5A’s might improve the SCC they provide. Dentists’ willingness and their confidence, which was influenced by the amount of training they received, were found to be the strongest correlating factors to initiate the cessation practice. Hence, more training on smoking cessation strategies are needed, including formal training in dental school’s curriculum [21]. In Israel, the IDA arranged seven training workshops for dentists from 2016 to 2019, aimed at enhancing their SCC practices (n =  ~ 200 dentists). Each workshop lasted for six hours, with participation being voluntary. At the end of each workshop, participants received a certificate for completing the smoking cessation training course for dentists. Initially, each session had approxmitately 20 dentists. Subsequently, attendance increased, with around 40–50 dentists attending each workshop.

While the 5A’s is the most accepted model for brief interventions, it has its limitations, with low levels of adoption in routine dental care [22], due to dental professionals’ lack of time and skills in tobacco use prevention [13]. A range of surveys conducted in the United States [11, 23, 24], Finland [25], Australia [2, 26] and Canada [27] indicated that the level of involvement declined as the dental professionals moved through the 5A’s protocol. The model’s first 2A’s—Ask and Advice—are most frequently carried out in interventions [21]. In this case, providing just brief advise (BA), which refers to the 2A’s might be a more feasible first step in dental settings [28].

This study aimed to determine: a) the extent to which Israeli dentists adhere to the 5A’s SCC guidelines; b) their knowledge and attitudes regarding SCC provision; and c) the factors that influence their provision of SCC.

## Methods

### Study design, participants and recruitment

An online cross-sectional survey was conducted in June–August 2020 in Israel. Participants were Israeli dentists who practiced clinical dentistry at the time of the survey. Pediatric dentists (pedodontists) were excluded from the study since most of their patients are unlikely to be smokers. The questionnaire was distributed by email (with two reminders) to all members of the Israeli Dentist Association (IDA), and to dentists who participated in past conferences who consented to receive additional marketing. The questionnaire was also distributed through social media in Facebook and WhatsApp groups for dentists. As an incentive, participants who completed the survey were invited to partake in a raffle for one tablet.

### Survey instrument

The study’s questionnaire (supplemental file 1) was based on the Theoretical Domains Framework (TDF) [22, 29, 30]. The TDF is a validated and integrative theoretical framework that covers 14 different domains that can be used to fully explore the behavior of healthcare professionals [22, 29, 31, 32]. It has been previously used extensively to determine difficulties of implementing SCC in various healthcare settings [31, 33, 34], including dental clinics [35].

The questionnaire was divided into four sections:

1. *Sociodemographic, smoking, and professional characteristics*, such as age, sex, smoking status, type of specialty, years in practice and as a specialist, workplace, and attendance of SCC training workshops.
2. *Knowledge regarding SCC*, using ten true/false/don't know questions. A composite score of correct answers was calculated from 0 to 10, with “don’t know” counted as incorrect.
3. *Attitudes regarding the provision of SCC*, using thirteen statements based on the TDF [22, 29], ranked on a 5-point Likert scale (strongly disagree [1] to strongly agree [5]). We included questions relating to 10 domains from the TDF, in order to maintain a reasonable questionnaire length. We selected the domains that we considered most important: knowledge, skills, role, beliefs about capabilities, optimism, beliefs about consequences, reinforcement, environmental context and resources, social influences and emotion.
  The rankings were averaged to generate a parametric measure of a mean composite attitude score. Scores were inverted for statements with negative language, to keep the directionality of the survey. In a separate analysis, rankings were also dichotomized to “agree”—(score [4] or [5]), or “disagree” (score [1] or [2] or [3]).
4. *5A’s performance*, assessing how frequently dentist performed each step of the 5A’s on an ordinal 5-point Likert scale (from never [1] to always [5]). “Assist” and “Arrange” were determined as a composite of several questions (two for “Assist” and three for “Arrange”), reflecting different ways in which the steps could be performed, with the highest-ranking response determining the final score for each respective step. These steps of the 5A’s were then combined into two different composite scores, which defined the 5A’s performance on a binary scale: (1) “Performing all of the 5A’s often and always” with participants who ranked all statements of the five steps as 4 or 5 being categorized as “yes”, and a score of 3 or lower for any of the steps categorized as “no”. (2) “Performing ‘Ask and Advise’ always” with participants who ranked both statements of the first two steps as 5 being categorized as “yes” and a score of 4 or lower for either of the steps categorized as “no”.

### Statistical analyses

Analysis was carried out using IBM SPSS Statistics, version 25 (IBM SPSS, Armonk, NY, USA). All analyses were done using 2-tailed p-value.

Descriptive analyses of sociodemographic, smoking, and professional characteristics, knowledge and attitude scores and SCC performance were conducted using frequency distributions for categorical variables and means with standard deviations (SD) for continuous variables.

Specialists were analysed as a single group, with all sub-specialties combined, due to the small number of participants within each sub-specialty. This prevented separate analyses by specific specialty type. Bivariate analyses were performed to assess differences between the dichotomous SCC scores (“Performing all of the 5A’s often and always” and “Performing ‘Ask and Advise’ always”) compared to not performing these. Continuous parametric analyses included independent t tests. Nonparametric analyses included Wilcoxon–Mann–Whitney tests for continues variables with a non-normal distribution, Chi-square tests for categorial variables and Fisher’s exact 2-sided test for dichotomous variables. Variables which were significant at a p < 0.05 in the bivariate analyses, in addition to age and sex which were treated as universal confounders, were entered into a multivariable logistic regression. We suspected an interaction between workshop participation and the composite attitude score. Therefore, we included an interaction variable in the regression model and calculated p-value for interaction (p_int_) and stratified the analysis by workshop participation.

### Ethics

The study received approval from the Ethical Committee at the Faculty of Medicine (approval #24062020). All the perticipants signed an electronic consent from.

## Results

Overall, n = 443 dentists responded, and of these, 410 (92.6%) were eligible and included in the final sample. N = 33 (7.4%) were excluded: 10 did not provide consent, 16 were pedodontists, 5 retirees, and 2 dentists residing outside of Israel.

Among the 410 eligible respondents, 251 (62.0%) were male. The mean age of the participants was 49.9 (SD 12.4). The majority never smoked cigarettes or used other nicotine or tobacco products (n = 258, 62.9%). Less than a fifth (n = 52, 12.8%) currently smoked cigarettes, with very few (6.1%, n = 25) currently using other products (primarily hookah, electronic cigarettes and cigars). Of the entire sample, only 1.7% (n = 7) reported currently using both cigarettes and other tobacco products. More than a third of the sample were specialists (n = 157, 38.3%). Most primarily worked in the private sector (n = 287, 70.3%) and had not participated in a SCC training workshop (n = 354, 86.3%). Sociodemographic, smoking characteristics and professional characteristics of the entire sample (n = 410) are presented in Table 1.

**Table 1:** Sociodemographic, smoking and professional characteristics of the total sample (n = 410) and by SCC performance

Variable	Total N = 410	Performing all 5A’s, often or always n = 405*			Performing Ask and Advise, always n = 408		
		Yes n = 57 (14.1%)	No n = 348 (85.9%)	p value	Yes n = 139 (34.1%)	No n = 269 (65.9%)	p value
Age (mean, SD)	49.86 (12.41)	49.60 (10.07)	49.79 (12.79)	0.898^a^	49.83 (12.19)	49.85 (12.56)	0.985^a^
Male sex (n, %)	251 (62.0%)	30 (53.6%)	216 (62.8%)	0.236^b^	79 (57.2%)	170 (64.2%)	0.195^b^
Years in practice (mean, SD)	22.64 (12.75)	22.39 (9.79)	22.58 (13.22)	0.898^a^	22.60 (12.23)	22.65 (13.06)	0.973^a^
Specialist (n, %)^c^	157 (38.3%)	31 (54.4%)	126 (36.2%)	0.012^b^	66 (47.5%)	91 (33.8%)	0.010^b^
Years as specialist (mean, SD)^d^	16.88 (12.01)	13.25 (8.94)	17.65 (12.52)	0.049^a^	15.71 (10.17)	17.76 (13.30)	0.307^a^
Primary workplace—private practice (compared to not private. n, %)	287 (70.3%)	43 (75.4%)	241 (69.7%)	0.435^b^	101 (72.7%)	185 (69.3%)	0.494^b^
Training workshop participation (n, %)	56 (13.7%)	15 (26.3%)	41 (11.8%)	0.006^b^	30 (21.6%)	26 (9.7%)	0.001^b^
Smokes cigarettes				0.354^e^			0.007^e^
Never (n, %)	273 (67.1%)	41 (71.9%)	228 (66.1%)		107 (77.0%)	164 (61.7%)	
Past (n, %)	82 (20.1%)	12 (21.1%)	69 (20.0%)		21 (15.1%)	61 (22.9%)	
Current (n, %)	52 (12.8%)	4 (7.0%)	48 (13.9%)		11 (7.9%)	41 (15.4%)	
Use of other smoking/Tobacco products^f^				0.153^e^			0.024^e^
Never (n, %)	341 (83.2%)	52 (91.2%)	285 (81.9%)		124 (89.2%)	215 (79.9%)	
Past (n, %)	44 (10.7%)	2 (3.5%)	41 (11.8%)		7 (5.0%)	37 (13.8%)	
Current (n, %)	25 (6.1%)	3 (5.3%)	22 (6.3%)		8 (5.8%)	17 (6.3%)	
Any Smoking/Tobacco use—ever user (n, %)	152 (37.1%)	19 (33.3%)	132 (37.9%)	0.557^b^	37 (26.6%)	115 (42.8%)	0.002^b^
Knowledge score (mean, SD)^g^	2.58 (1.51)	3.07 (1.64)	2.51 (1.47)	0.008^a^	2.60 (1.50)	2.57 (1.52)	0.726^a^
Attitude score (mean, SD)^h^	2.65 (0.60)	3.13 (0.58)	2.57 (0.56)	< 0.001^i^	2.83 (0.60)	2.56 (0.57)	< 0.001^i^

*Missing data: 5A’s performance (n = 5), ask and advise (n = 2), sex (n = 5), cigarette smoking status (n = 3), any smoking/tobacco use (n = 3), years in practice (n = 1), primary workplace (n = 2), knowledge score (n = 9), composite attitude score (n = 7)

^a^Independent t test

^b^Fisher’s exact 2-sided test

^c^The most prevalent specialty (n = 64, 40.8%) was Periodontics, followed by Maxillofacial surgery (n = 26, 16.6%), Prosthodontics (n = 21, 13.4%), Endodontics (n = 18, 11.5%) and Orthodontics (n = 14, 8.9%)

^d^For the n = 157 specialists

^e^Chi-square tests

^f^Other tobacco products included: hookah, electronic cigarettes, cigars and heated tobacco products

^g^Knowledge score on a scale from 1 to 10

^h^Composite attitude score on a scale from 1 to 5

^i^Wilcoxon–Mann–Whitney test

### Knowledge and attitudes

**Fig. 1:**
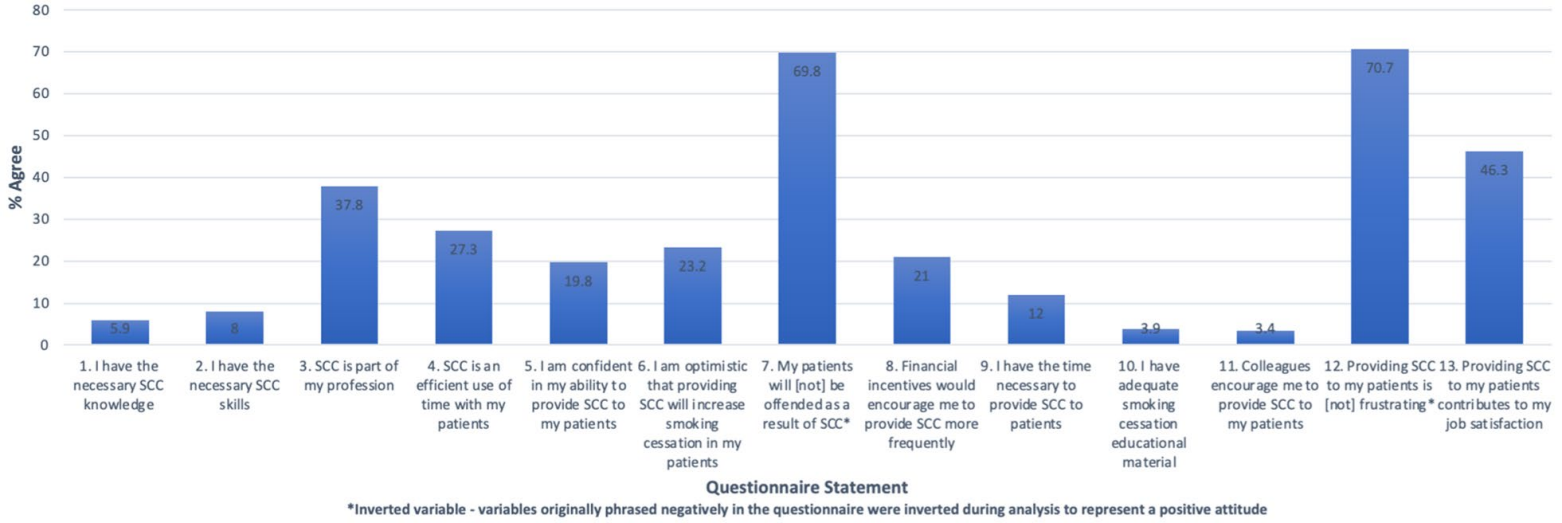
% Agreement with positive statements regarding SCC

The mean knowledge score was 2.58 (out of 10), SD = 1.51. The mean attitude score was 2.65 (out of 5), SD = 0.60. Rates of agreement for each TDF attitude are presented in Fig. 1 (n = 410). Overall, participants tended to display negative attitudes in most of the domains. However, 70.7% (n = 290) did not believe that providing SCC to their patients is frustrating and 69.8% (n = 286) agreed that providing SCC would not offend their patients. The performance of specialists and non-specialists in agreeing with positive statements regarding SCC is presented in the Supplemental file 2 (Fig. 1).

### Performance of all the 5A’s “Often and Always”

**Fig. 2:**
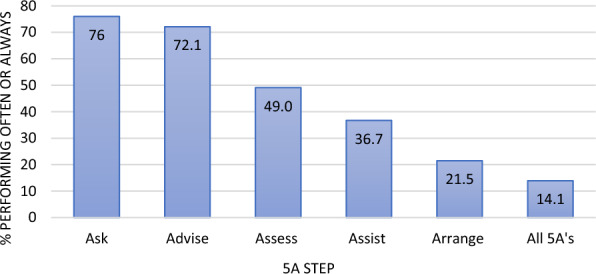
Percent performing each step of the 5A’5, often or always

Overall performance of all 5A’s was low with only 57/405 participants (14.1%) reporting completing all the 5A’s ‘often or always’ (Fig. 2). While 76.0% (n = 310/408) of dentists perform ‘Ask’ and 72.1% (n = 294/408) perform ‘Advise’, considerably less dentists performed the following three steps, with performance reduced with each step—49.0% (n = 200/408) performed ‘Assess’, 36.7% (n = 150/408) performed ‘Assist’, and 21.5% (n = 88/408) performed ‘Arrange’. The performance of specialists was significantly higher compared to non-specialists in all steps of the 5A’s ‘often or always’, p < 0.05 (Supplemental file 2: Fig. 2).

Among those who performed all the 5A’s ‘often or always’, a higher rate of participants were specialist (including: Periodontics, Maxillofacial surgery, Prosthodontics, Endodontics and Orthodontics) (n = 31/57, 54.4%), compared to the non-performers group, where only 36.2% (n = 126/348) were specialist, p = 0.012 (Table 1). Participants with fewer years as specialists demonstrated a statistically higher performance of the 5A’s (mean = 13.25, SD 8.94) compared to those with more years as specialists (mean = 17.65, SD 12.52; p = 0.049). Training workshop participants exhibited a statistically higher rate of 'often or always' performing all the 5A's (n = 15, 26.3%) compared to non-performers (n = 41, 11.8%; p = 0.006). The mean knowledge score (3.07, SD 1.64) and composite attitude score (3.13, SD 0.58) were higher among participants who ‘often or always’ performed all the 5A’s, compared to non-performers (p = 0.008 and p < 0.001, respectively). No difference was found between the participants whose primary workplace was the private practice and performed all the 5A’s ‘often or always’ (n = 43/284, 15.1%), compared to those whose primary workplace was not private (n = 14/119, 11.8%), p = 0.435.

Table 2 provides the results of the multivariate analyses for factors associated with ‘often and always’ performing all 5A’s. There was a significant interaction between workshop participation and the composite attitude score (p_int_ = 0.018). Therefore, we present the full model with the interaction variable, and stratified by workshop participating in the workshop.

**Table 2:** Logistic regression 5A performance, n = 405

Variable	Often or always performing all 5A’s n (%)	Crude (univariable)		Adjusted model with interaction variable*		Stratified analysis by workshop participation			
						Workshop participation—Yes		Workshop participation—No	
		OR (95% CI)	p-Value	OR (95% CI)	p-Value	OR (95% CI)	p-Value	OR (95% CI)	p-Value
Specialist: No (n = 248)	26 (10.5%)	Ref		Ref					
Specialist: Yes (n = 157)	31 (19.7%)	2.10 (1.19, 3.70)	0.010	2.01 (1.11, 3.66)	0.022	2.57 (0.63, 10.40)	0.187	2.13 (1.03, 4.42)	0.042
Knowledge score^		1.25 (1.05, 1.49)	0.010	1.19 (0.98, 1.445)	0.076	0.87 (0.53, 1.45)	0.600	1.15 (0.91, 1.46)	0.240
Attitude score^		5.97 (3.36, 10.60)	< 0.001			12.89 (2.06, 80.69)	0.006	5.64 (2.92, 10.89)	< 0.001
Workshop* attitude (interaction)					0.018				

*Model was adjusted to age and sex. *CI* confidence interval. *n* number

^Knowledge score (0–10) and attitude score (1–5): the odds ratio (OR) represents the change in odds of the outcome for each one-unit increase in the respective scores

Specialists had better odds of ‘often or always’ performing the 5A’s (adjusted Odds Ratio (OR) = 2.01, p = 0.022). Knowledge score was not associated with performance in any of the adjusted models. Attitude score was significantly associated with performance in both workshop participants and non-participants. However, the association was stronger among workshop participants (OR = 12.89, 95% Confidence Intervals (CI) 2.06–80.69, p = 0.006) than among non-participants (OR = 5.64, 95% CI 2.92–10.89, p < 0.001).

### Performance of Ask and Advise “Always”

Overall, 34.1% (n = 139) of participants reported ‘always’ performing ‘Ask’ and ‘Advise’ (Table 1). Participants who never smoked exhibited a higher rate of 'always' performing 'Ask and Advise' (n = 107, 77%, p = 0.007). A similar association was observed for users of other smoking/tobacco products, with 89.2% (n = 124) of performers reporting never using them (p = 0.024), and 'ever users' displaying a significantly lower rate of 'always' performing 'Ask and Advise' (n = 37, 26.6%) compared to not performing (n = 115, 42.8%), p = 0.002. Specialists and workshop participants also demonstrated higher rates of ‘always’ performing 'Ask and Advise' (n = 66, 47.5%, p = 0.010, and n = 30, 21.6%, p = 0.001, respectively). Additionally, participants who 'always' performed 'Ask and Advise' exhibited a higher composite attitude score (mean = 2.83, SD 0.60) compared to non-performers (mean = 2.56, SD 0.57), p < 0.001.

Logistic regression results for ‘always’ performing ‘Ask and Advise’ is depicted in Table 3. Specialists had better odds of ‘always’ performing ‘Ask and Advise’ (adjusted OR = 1.71, p = 0.022). Participants with more positive attitudes also performed better (adjusted OR = 2.48, p < 0.001). The OR of ‘always’ performing ‘Ask and Advise’ was about half in ever smokers (or tobacco users) compared to never smokers (adjusted OR = 0.48, p = 0.004). Workshop participation was not significant in the adjusted regression (p = 0.07).

**Table 3:** Logistic regression ask advise performance, n = 408

Variable	‘Always’ performing ‘Ask and Advise’ n (%)	Crude (univariable)		Adjusted* (multivariable)	
		OR (95% CI)	p-value	OR (95% CI)	p-value
Specialist					
No (n = 251)	73 (29.1%)	Ref^	0.007	Ref^	0.022
Yes (n = 157)	66 (42.0%)	1.77 (1.16, 2.69)		1.71 (1.08, 2.72)	
Workshop					
No (n = 352)	109 (31.0%)	Ref^		Ref^	
Yes (n = 56)	30 (53.6%)	2.57 (1.45, 4.56)	0.001	1.76 (0.95, 3.27)	0.072
Smoking/other Tobacco use					
Never user (n = 256)	102 (39.8%)	Ref^		Ref^	
Ever user (n = 152)	37 (24.3%)	0.49 (0.31, 0.76)	0.002	0.48 (0.30, 0.79)	0.004
Attitude score		2.26 (1.56, 3.27)	< 0.001	2.48 (1.66, 3.70)	< 0.001

^Ref—reference group. *CI* confidence interval, *n* number. *Model was adjusted to age and sex

## Discussion

In this study, participating Israeli dentists had low levels of knowledge and attitude scores regarding SCC provision, and were not implementing SCC as per the accepted guidelines from other countries [16–19].

Only 14.1% of the sample performed all of the 5A’s ‘often or always’. In a study of general dentists in the USA, 39% of respondents (n = 265) reported assisting with quitting, but only 4% arranged follow-up [23]. Another narrative review found that a low percentage of dental professionals took action in arranging referrals, with rates ranging from 1 to 47% [21]. Our sample also displayed (Fig. 2) a pattern of reduced participation for each consecutive step of the 5A’s. Similar results of decline in the level of involvement through the 5A’, and lowest performance of the final steps are seen in other studies elsewhere [11, 21, 36–38]. The low numbers come from self-report of 5A’s performance and are likely to be an overestimation. As each additional 5A’s step requires more time and involvement from the dentist, this trend is not surprising. It is also possible that some of the participants in our study did not have any SCC training throughout dental school (to the best of our knowledge, formal training in smoking cessation is not routinely provided in dental schools in Israel). Hence, they were unaware of the concept of the 5A’s. In a survey conducted on Florida dentists, 88% of the 1232 respondents stated that they were not familiar with these guidelines [38]. In a survey of dental school curricula on tobacco education across 21 European countries, 67% of dental schools (n = 45) reported implementing tobacco education in their curriculum. However, only 40% (n = 18) included practical skills training for their students [39].

Our main outcome was performance of all the 5As’s. However, considering the low implementation rates we found in the literature, we also investigated “brief advice”: ‘Ask and Advise’, as a secondary outcome. ‘Ask and Advise’ alone is generally considered cheaper and less time consuming, compared to the 5A’s [28]. We consider ‘Ask and Advise’ to be the most basic level of SCC that all dentists should always provide their patients. Therefore, we used a stricter threshold and only considered dentists who report ‘always’ performing ‘Ask and Advise’. The performance of both ‘Ask and Advise’ (34.1%) appeared to be low using this metric. This is opposed to our results of ‘often or always’ performing ‘Ask and Advise’ which were generally high (76.0% and 72.1%. respectively). Other surveys demonstrate a wide range of percentages of dentists routinely performing ‘Ask and Advise’ [21, 23, 27, 36, 38]. The high ‘often and always’ performance of our sample could be, in part, attributed to the relatively high percentage of specialists among our participants (38%), exceeding that of the general dentist population (about 10%) [40]. Our results confirmed that specialists were better at providing SCC to their patients than non-specialists. This could be attributed to their experience, additional training improving their SCC skills during residency and their attitudes towards SCC.

Attitude score, as determined by the TDF, was found in our study to be an important predictor of 5A’s. Our results demonstrate that dentists in Israel seem to acknowledge that SCC is important, but they do not feel capable of providing SCC to their patients. As workshop attendance was associated with better SCC, and an interaction was identified between workshop participation and attitude score, positive attitudes was more strongly associated with performance among workshop participants compared to nonparticipants. This could be explained by attitudes influencing workshop participation which in turn influences better performance. Alternatively, dentists with more positive attitudes toward SCC and better performance might be more willing to participate in SCC training workshops. Nonetheless, it is crucial to incorporate SCC in clinical training and adapt dentists’ work settings to allow for its implementation. While time may be difficult to find, introducing tools such as smoking cessation educational materials could be a feasible first step.

Our study has limitations, including its cross-sectional design, which precludes causal inferences. The voluntary questionnaire may introduce selection bias, as it relies on dentists who choose to respond, potentially skewing the sample. Targeting dentists from conventions and social media may further contribute to selection bias. Additionally, the IDA has over 5,000 members (according to their website), but it is possible that not all dentists in Israel are IDA members, which could limit the generalizability of our sample to the entire dentist population in Israel. To mitigate this, we also distributed the questionnaire through social media groups for dentists and included dentists who participated in previous conventions.

The high proportion of specialists in our sample could lead to an overestimation of SCC, weakening our results. Yet, other demographic characteristics were similar to the general dentist population in Israel [40]. Furthermore, the survey was conducted during the second wave of COVID-19, which may have affected the participation of dentists.

Our study is susceptible to recall and social desirability biases due to self-report questions. In a context of a SCC questionnaire, participants could feel embarrassed about personal smoking history and not report it truthfully. Nevertheless, self-report questionnaires save time and are cost efficiency, reaching a broad and diverse population. Hence, they serve as a preferred tool for research data collection [41, 42].

### Implications for policy and practice

Future training programs and interventions should be developed, considering dentists' perceived barriers and facilitators of providing SCC. The IDA, together with the Dental Health Division and the Health Education and Promotion Department at the Ministry of Health, could initiate the development of guidelines recommending the use of the 5A’s, beginning with an abridged version (two or three steps like ask, advise, and arrange) for less motivated dentists. Encouraging automatic referral systems to support services, like the national Quitline, might overcome time constraints. Integrating smoking status into electronic charts and defining SCC performance as a quality metric could help establish a supportive environment.

Health Maintenance Organizations (HMOs) and the Ministry of Health should play a more active role in SCC training, leveraging their expertise in continuing education and training for dentists. Collaboration with health promoters within HMOs could increase the uptake of workshops and medication-based treatments, thereby raising cessation rates. Moreover, HMOs and other institutions should hold dentists accountable for performing SCC, fostering a culture of responsibility. Early integration of SCC habits during dental education and residency is crucial for sustaining these practices in public and private practice settings.

Future research should also examine whether patients who received counseling from dentists followed through with the advice and whether it led to successful cessation. This would provide further insight into the effectiveness of SCC in dental settings and help refine these interventions.

## Conclusions

This study highlights the insufficient performance of SCC among Israeli dentists, revealing gaps in knowledge and attitudes related to SCC and adherence to the 5A's model. Various measures, such as training, automatic referral systems, and integrating SCC as quality measures, alongside active involvement from HMOs and the Ministry of Health, may improve SCC provision among Israeli dentists.

## Supplementary Information

Additional file 1.

Figure 1. Percentage of Specialists and Non-Specialists Agreeing with positive statements regarding SCC.

Figure 2. Percentage of Specialists and Non-Specialists Performing Each Step of the 5A's, Often or Always.

## Data Availability

The datasets used and/or analysed during the current study are available from the corresponding author on reasonable request.

## Abbreviations

SCC: Smoking cessation care
TDF: Theoretical domains framework
IDA: Israeli Dental Association
ADA: American Dental Association
BA: Brief advise
SD: Standard deviation
CI: Confidence intervals
OR: Odds ratio
USA: Unites States of America
UK: United Kingdom
HMOs: Health maintenance organizations
