# Supplementary material for: Israeli dentists’ knowledge, attitudes, and practices regarding smoking cessation care
Source: Isr J Health Policy Res. 2024 Nov 11;13:66. doi: 10.1186/s13584-024-00653-5 (PMC11552324; doi:10.1186/s13584-024-00653-5)
Supplement: Supplementary file 1 — Additional file 1. [file 13584_2024_653_MOESM1_ESM.docx]

Supplemental file 1 – Study’s questionnaire

**Knowledge, attitudes and practices among Israeli dental professionals regarding smoking cessation care**

We are conducting a survey on knowledge, attitudes, and practices regarding smoking cessation support for patients among dental professionals. This is an anonymous survey sent via email. As researchers, we do not have access to any personal data of the participants. The work is part of a thesis for a master's degree in public health. All data will be kept confidential and used for research purposes only. You are free to choose not to answer any questions in the survey and can stop responding at any time. The estimated time to complete the questionnaire is approximately 30 minutes.

Responding to the survey indicates your consent to participate.

To thank you for your participation, at the end of the questionnaire, you will be invited to participate in a raffle for a tablet. If you choose to participate in the raffle, you will be directed to a separate form after submitting the questionnaire.

We appreciate your collaboration.

Dr. Yael Bar-Zeev, Senior Lecturer, School of Public Health, Faculty of Medicine, The Hebrew University

Dr. Itzhak Tayeb, Specialist in Periodontology and Director of the Specialist Dental Clinic, Hadassah Mount Scopus University Hospital

Ms. Tamar Rosenberg, Second-year student in Public Health, Faculty of Medicine, The Hebrew University

*The Israel Dental Association (IDA) informs that, aside from consenting to the distribution of the online questionnaire via email to members of the IDA, the IDA bears no responsibility, of any kind, regarding the distributed questionnaire, its content, the survey conducted, and all related matters. The sole responsibility lies with the initiators and/or formulators of the online questionnaire and/or anyone conducting or participating in the survey.

1. Do you agree to participate in the study?

Yes/no

1. What is your sex?

Female/male

1. What is your age?
2. What’s the year when you received your license to practice dentistry?
3. Are you a specialist?

Yes/no

1. If you answered ‘yes’ to question 5: what’s the year you received your specialty certification?
2. If you answered ‘yes’ to question 5: What’s your specialty?

Oral and maxillofacial surgery

Pediatric dentistry

Orthodontics

Periodontics

Community dentistry

Oral medicine

Oral and maxillofacial pathology

Prosthodontics

Restorative dentistry

Other (specify):

1. Mark all the places where you work:

Health Maintenance Organization (HMO)

Private Practice

Hospital

Other

1. What’s your primary place of work?

Health Maintenance Organization (HMO)

Private Practice

Hospital

Other

1. Do you smoke cigarettes?

Yes, daily

Yes, sometimes

No, I used to smoke

No, never

1. Do you use other tobacco or smoking products (e.g. hookah, e-cigarette, heated tobacco products, cigars, chewing tobacco?

Yes, daily

Yes, sometimes

No, I used to use )any of these products)

Never used any of these products

1. If you use any tobacco or smoking products, specify which (you can choose more than one answer)

Hookah

Chewing tobacco

Cigars

E-cigarette / vaporizer

Heated tobacco products e.g. IQOS

1. Did you ever attend a smoking cessation care (SCC) training workshop? Yes (specify)/ no
2. Did you attend Drs. Dahan and Afek’s SCC training workshops? Yes/no

If you answered ‘yes’ to question 14, circle (1 – very little, 5 – very much):

| 5 | 4 | 3 | 2 | 1 | 15. To what extent do you use the tools provided in the workshop? |
| --- | --- | --- | --- | --- | --- |
| 5 | 4 | 3 | 2 | 1 | 16. To what extent do you think you’ve improved your SCC you’re providing to your patients? |

Practices

17. How often do you (circle):

|  | **Never**  **(0%)** | **Seldom**  **(1-25%)** | **Sometimes**  **(26-50%)** | **Often**  **(51-75%)** | **Always**  **(76-100%)** |
| --- | --- | --- | --- | --- | --- |
| **Ask patients about smoking status?** | 1 | 2 | 3 | 4 | 5 |
| **Advise patients to stop smoking?** | 1 | 2 | 3 | 4 | 5 |
| **Assess how ready are patients to quit smoking?** | 1 | 2 | 3 | 4 | 5 |
| **Assist patients in quitting?** | 1 | 2 | 3 | 4 | 5 |
| **Prescribe a medication for smoking cessation?** | 1 | 2 | 3 | 4 | 5 |
| **Refer patients to consult with their general practitioner regarding smoking cessation?** | 1 | 2 | 3 | 4 | 5 |
| **Refer patients to other SCC providers (eg group therapy)?** | 1 | 2 | 3 | 4 | 5 |
| **Schedule a SCC follow-up visit?** | 1 | 2 | 3 | 4 | 5 |

Knowledge

18. Select true/false/don’t know:

| **Statement** | **True** | **False** | **Don’t know** |
| --- | --- | --- | --- |
| a. 30% of Israelis smoke |  |  |  |
| b. Nicotine substituents such as chewing gum and lozenges can cause a prickling sensation in one’s mouth |  |  |  |
| c. SCC workshops and medications aren’t included in the Israeli Ministry of Health health basket |  |  |  |
| d. Smoking inhibits dental postprocedural wound healing |  |  |  |
| e. Nicotine gums are to be chewed differently than regular gum |  |  |  |
| f. Epilepsy is a contraindication for Bupropion |  |  |  |
| g. Varenicline therapy should be initiated on the day of smoking cessation |  |  |  |
| h. Most of smoking cessation relapses happen on the first week |  |  |  |
| i. No more than one nicotine replacement threapy is to be taken at a time |  |  |  |
| j. In Israel, there is a free national quitline for smoking cessation |  |  |  |

Answer code:

1. false
2. true
3. false
4. true
5. true
6. true
7. false
8. true
9. false
10. true

TDF

19. Please indicate your agreement with the following phrases on a scale of 1 – strongly disagree to 5 – strongly agree:

| **TDF Domain** | Statement | Strongly disagree | Disagree | Partially agree | Agree | Strongly agree |
| --- | --- | --- | --- | --- | --- | --- |
| D1 Knowledge | 1. I have the necessary knowledge to support my patients in a smoking cessation process | 1 | 2 | 3 | 4 | 5 |
| D2 Skills | 2. I have the necessary skills to support my patients in a smoking cessation process | 1 | 2 | 3 | 4 | 5 |
| D3 Role | 3. Providing smoking cessation care is part of my professional role as a dentist | 1 | 2 | 3 | 4 | 5 |
| D3 Role | 4. Providing smoking cessation care is an efficient use of time during my appointment with patients | 1 | 2 | 3 | 4 | 5 |
| D4 Beliefs about capabilities | 5. I am confident in my ability to provide SCC to my patients | 1 | 2 | 3 | 4 | 5 |
| D5 Optimism | 6. I am optimistic that providing SCC will increase smoking cessation in my patients | 1 | 2 | 3 | 4 | 5 |
| D6 Beliefs about consequences | 7. My patients might be offended if I encourage them to quit smoking | 1 | 2 | 3 | 4 | 5 |
| D7 Reinforcement | 8. Financial incentives for the provision of SCC would encourage me to provide SCC more frequently | 1 | 2 | 3 | 4 | 5 |
| D11 Environmental context and resources | 9. I have the necessary time to adequately provide SCC to my patients | 1 | 2 | 3 | 4 | 5 |
| D11 Environmental context and resources | 10. I have adequate smoking and smoking cessation educational materials | 1 | 2 | 3 | 4 | 5 |
| D12 Social influences | 11. Dentistry colleagues encourage me to provide SCC to my patients | 1 | 2 | 3 | 4 | 5 |
| D13 Emotion | 12. It is frustrating for me to provide SCC to my patients | 1 | 2 | 3 | 4 | 5 |
| D13 Emotion | 13. Providing SCC to my patients contributes to my job satisfaction | 1 | 2 | 3 | 4 | 5 |

20. Do you want to participate in a raffle for a tablet?

Yes/no
