# Supplementary material for: Israeli dentists’ knowledge, attitudes, and practices regarding smoking cessation care
Source: Isr J Health Policy Res. 2024 Nov 11;13:66. doi: 10.1186/s13584-024-00653-5 (PMC11552324; doi:10.1186/s13584-024-00653-5)
Supplement: Supplementary file 2 — Figure 1. Percentage of Specialists and Non-Specialists Agreeing with positive statements regarding SCC. [file 13584_2024_653_MOESM2_ESM.docx]

**Supplemental file 2 - Figure 1**
