# Supplementary material for: Israeli dentists’ knowledge, attitudes, and practices regarding smoking cessation care
Source: Isr J Health Policy Res. 2024 Nov 11;13:66. doi: 10.1186/s13584-024-00653-5 (PMC11552324; doi:10.1186/s13584-024-00653-5)
Supplement: Supplementary file 3 — Figure 2. Percentage of Specialists and Non-Specialists Performing Each Step of the 5A's, Often or Always. [file 13584_2024_653_MOESM3_ESM.docx]

**Supplemental file 2 - Figure 2**
